# Two- and three-dimensional measurements of leg length change using an accelerometer-based portable navigation system in total hip arthroplasty

**DOI:** 10.1186/s12891-023-07006-4

**Published:** 2023-11-09

**Authors:** Masahiro Hasegawa, Yohei Naito, Shine Tone, Akihiro Sudo

**Affiliations:** Department of Orthopaedic Surgery, Mie University Graduate School of Medicine, 2-174 Edobashi, Tsu City, Mie 514-8507 Japan

**Keywords:** Navigation, Leg length, Two-dimensional, Three-dimensional, Total hip arthroplasty

## Abstract

**Background:**

The aim of this study was examining the accuracy of accelerometer-based portable navigation systems (HipAlign) when measuring leg length changes using two-dimensional (2D) and three-dimensional (3D) methods.

**Methods:**

Inclusion criteria were patients ≥ 20 years old with symptomatic hip disease who underwent primary total hip arthroplasty (THA) in the supine position using HipAlign between June 2019 and April 2020. The exclusion criteria were patients who underwent THA via a posterior approach. We examined correlations between the leg length change measurement with HipAlign and either 2D or 3D measurement. We performed a multivariate analysis to determine which factors may have influenced the absolute error results.

**Results:**

This study included 34 patients. The absolute error in leg length change between the HipAlign and 3D measurement (4.0 mm) was greater than the HipAlign and 2D measurement (1.7 mm). There were positive correlations between leg length change with HipAlign and 2D and 3D measurements. Male patients had larger errors with 2D measurement. No significant factors were identified for 3D measurement.

**Conclusion:**

HipAlign provided acceptable measurement accuracy for leg length changes.

## Background

Leg length discrepancy is a common concern following total hip arthroplasty (THA). Furthermore, it remains one of the most common reasons for litigation against the orthopedic community in the US [1]. To equalize leg length, surgeons perform preoperative planning using radiological templates or intraoperative measurement methods, including calipers, radiographs, and navigation systems [2–6].

Navigation systems have been used for accurate cup placement to reduce complications from cup malpositioning, including impingement and dislocation [7–9]. Using navigation systems, surgeons can also detect a change in leg length. Computed tomography (CT)-based navigation can be used; however, the associated high cost and radiation exposure are problematic [10]. Large console image-free navigation does not require preoperative CT but is expensive [11]. In recent years, portable navigation systems have been developed, which are lower in cost. The accuracy of acetabular cup placement using a portable navigation system (HipAlign, OrthAlign, Aliso Viejo, CA, USA) has been studied. Previous studies demonstrated improved accuracy of cup inclination and anteversion using navigation systems [12–16]. Only two studies have demonstrated the accuracy of leg length change measurements using HipAlign [17, 18]. These studies examined measurement accuracy using 2D radiographs. To the best of our knowledge, there are no studies that examine measurement accuracy using 3D evaluation. The purpose of this study was to examine the accuracy of leg length change measurements by the portable navigation system (HipAlign) using 3D evaluation. Comparisons were also made between the 2D and 3D evaluations. To ensure accurate 2D and 3D measurements, we previously performed a reliability study using the intra-class and inter-class correlation coefficients [19, 20]. The intra-class and inter-class correlation coefficients for the 2D measurements were 0.98 and 0.92, respectively. The intra-class and inter-class correlation coefficients for the 3D measurements were 0.97 and 0.94, respectively [19].

We hypothesized that 2D evaluation would show better accuracy for leg length change using HipAlign.

## Materials and methods

### Patients

Inclusion criteria were patients ≥ 20 years old with symptomatic hip disease who underwent primary THA in the supine position under general anesthesia using HipAlign between June 2019 and April 2020. The exclusion criteria were patients who underwent THA via a posterior approach, such as hips with a high degree of dislocation (Crowe groups 3 and 4) [21] requiring subtrochanteric osteotomy and revision hip arthroplasty.

**Fig. 1:**
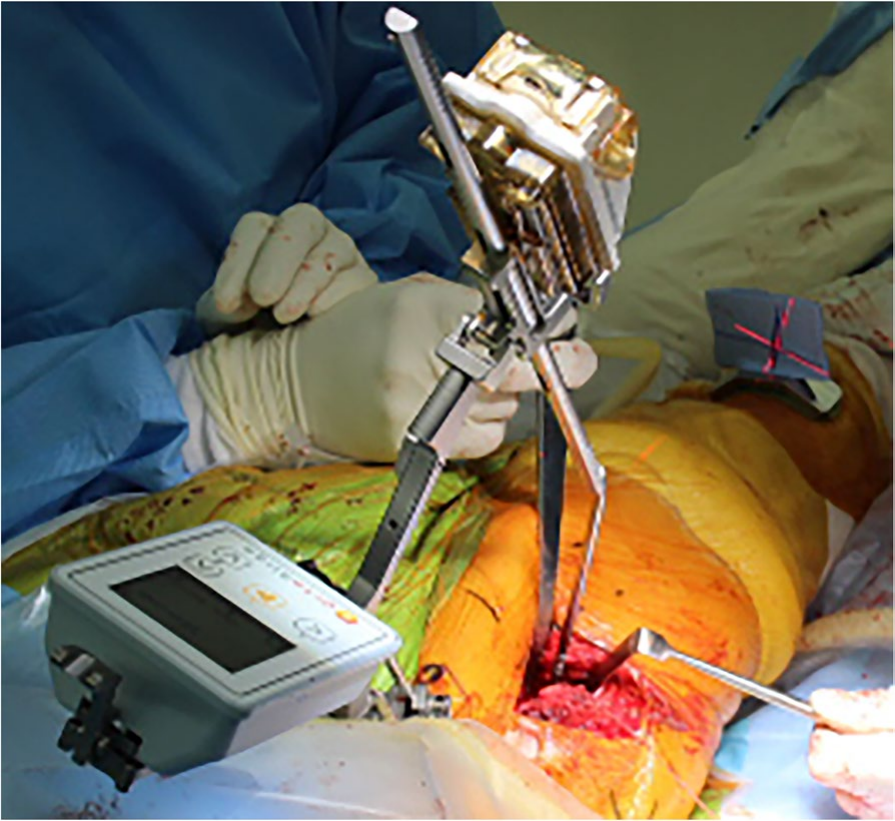
Sensor with monitor and probe of HipAlign were attached to the pelvis. Laser projection aligned with the marking on the vertical target

**Fig. 2:**
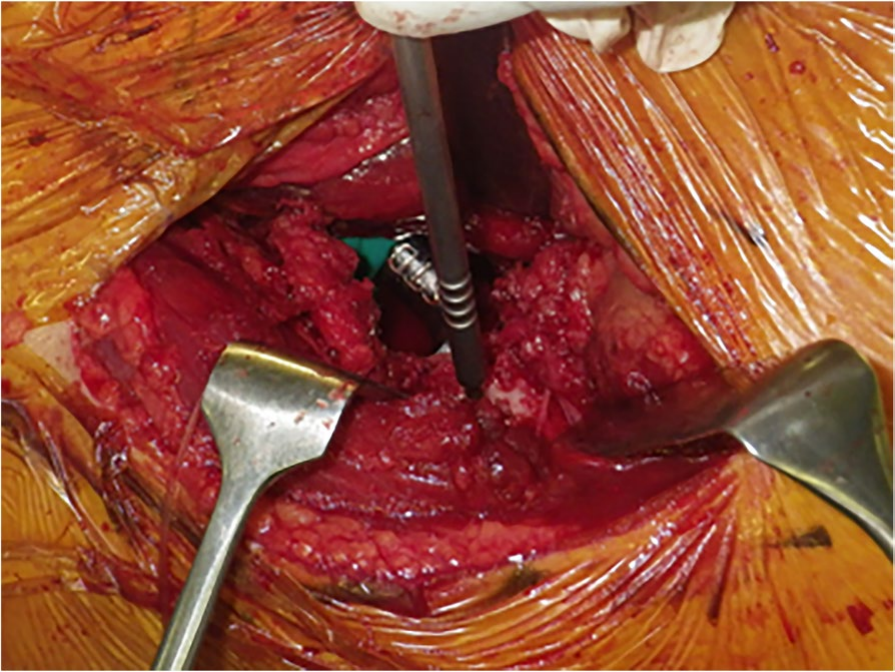
A registration probe was placed in the hole at the proximal femur

All hips were implanted with cementless components by the same surgeon (MH). The hips were exposed via a direct anterior approach (DAA) on a traction table or with a modified Watson-Jones approach (anterolateral supine approach: ALS), with the patient in a supine position. Twenty-six patients were treated using DAA, and the remaining eight patients were treated using ALS. ALS was selected in cases with severe deformity or excessive anteversion of the femoral neck. Two pins, 4.5 mm in diameter, were placed at the iliac crest, and the navigation unit was fixed to these pins. The location of the bilateral anterior superior iliac spine (ASIS) and pubic symphysis were recorded. HipAlign can measure the leg length change using a femoral registration screw, probe, laser module, and thigh plate for a vertical laser target (Fig. 1). Prior to neck osteotomy, a small reference screw was placed in the proximal femur for femoral registration. However, it can be difficult to fix this screw to the proximal femur in patients with DAA or ALS. A hole was made using a 2.0 mm diameter Kirschner wire at the proximal femur, and a registration probe was placed in this hole (Fig. 2). For intraoperative leg length measurement, the laser module was attached to the bracket of the navigation unit and the thigh plate was placed on the distal anterior thigh. A vertical laser target was attached to the thigh plate. The limb was positioned in the neutral reference position, and the laser projection on the vertical target was traced using a marking pen [18]. The surgeon realigned the vertical laser target to the laser projection and registered the marker (Fig. 1).

**Fig. 3:**
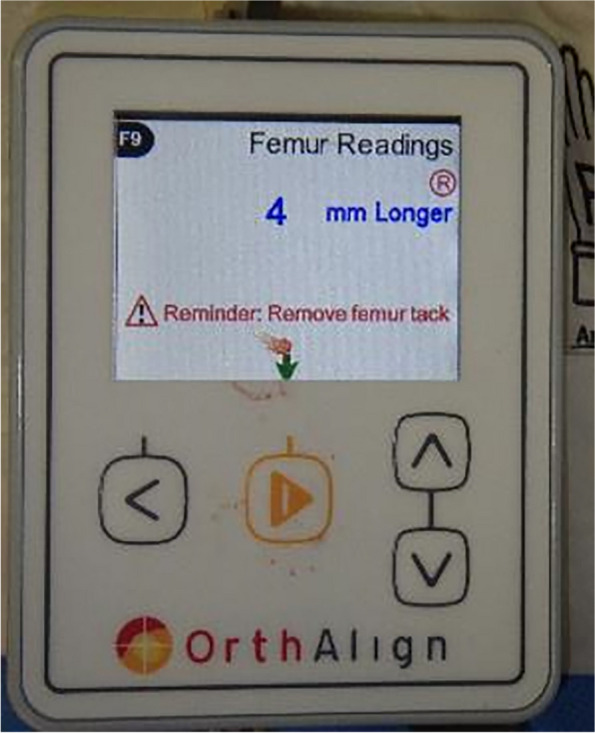
Monitor of HipAlign showing 4 mm longer

Leg length change was defined as the change in length measured before the neck cut and after the reduction with the same six hip directions (flexion/extension, abduction/adduction, and external rotation/internal rotation). Surgeons can detect 2D leg length changes on the monitor (Fig. 3). A G7 PPS Finned BoneMaster Limited Hole Shell (Zimmer Biomet, Warsaw, IN, USA) was used. Regarding stems, Taperloc Microplasty stems (Zimmer Biomet, Warsaw, IN) or AMIStem (Medacta, Castel San Pietro, Switzerland) were used. CT was performed preoperatively from the pelvis to knee joint. Neck length was determined according to preoperative planning using 3D software (ZedHip, LEXI Co. Ltd, Tokyo, Japan). If instability after trial reduction was found, the surgeons selected a longer neck than was decided in preoperative planning.

### Evaluation

The anteroposterior radiographs of the hips were performed in the supine position preoperatively and postoperatively. The leg length discrepancy on 2D measurement was measured as the distance between the horizontal line connecting the bilateral tear drops and the medial apex of the lesser trochanter. Leg length change on 2D measurement was defined as the difference between the preoperative and postoperative leg length. The leg length change on 2D measurement was subtracted from the leg length discrepancy pre-and -post-surgery. CT was performed from the pelvis to the knee preoperatively and 2 weeks postoperatively. The ZedHip was used to examine pre- and postoperative leg length discrepancies and compared with 3D measurement, which was assessed as the distance from the ASIS to the intercondylar fossa of the femur. Pre- and 2 weeks postoperative CT digital images were superimposed using the ZedHip. The leg length change measured by ZedHip was subtracted from the leg length discrepancy pre-and -post-surgery. To evaluate the accuracy of HipAlign based on 2D and 3D measurements, intraoperative leg length change was compared with 2D and 3D measurements [19]. Hip measurements with absolute error values within 3, 5, and 10 mm were determined using 2D and 3D measurements. All patients were followed up post-THA and examined for potential complications.

### Statistical analysis

Power analysis was performed, and a minimum of 27 patients were required to provide appropriate power (β = 0.80) at a significance level of 0.05. The absolute errors in leg length change were compared between the 2D and 3D measurements using the Wilcoxon signed-rank test. Correlation analyses were performed using Spearman’s rank correlation test. The correlations between leg length change measured with HipAlign and 2D or 3D measurements were evaluated. The correlation between leg length change measured using 2D and 3D measurements were determined. Multivariate analysis was performed to determine the factors affecting absolute errors, including age (≥ 65 years and < 65 years), sex, BMI (≥ 25 kg/m^2^ and < 25 kg/m^2^), approach, and Crowe group. *P*-values of < 0.05 were considered statistically significant. Data were statistically analyzed using the EZR software program, version 1.61 (Saitama Medical Center, Jichi Medical University, Saitama, Japan) [22].

## Results

This study included 34 patients. There were 3 men and 31 women, with a mean age of 66.9 years (range, 48–82 years) and mean body mass index (BMI) of 23.4 kg/m^2^ (range, 18.9–28.2 kg/m^2^). The preoperative diagnosis for all patients was osteoarthritis of the hip. Thirty hips were classified as Crowe group 1 [21]. Four hips were classified as Crowe group 2 [21].

The absolute error for leg length change between the HipAlign measurement and 2D measurement was 1.71 ± 1.92 mm (range, 0–5 mm). These errors were within 3 mm for 79% of patients (27 of 34 patients), and the remaining patients had errors within 5 mm.

The absolute error in leg length change between HipAlign measurement and 3D measurement was 4.03 ± 3.51 mm (range, 0.4–17.7 mm). These errors were within 3 mm for 53% of patients (18 patients), within 5 mm for 82% of patients (28 patients), and within 10 mm for 94% of patients (32 patients).

**Fig. 4:**
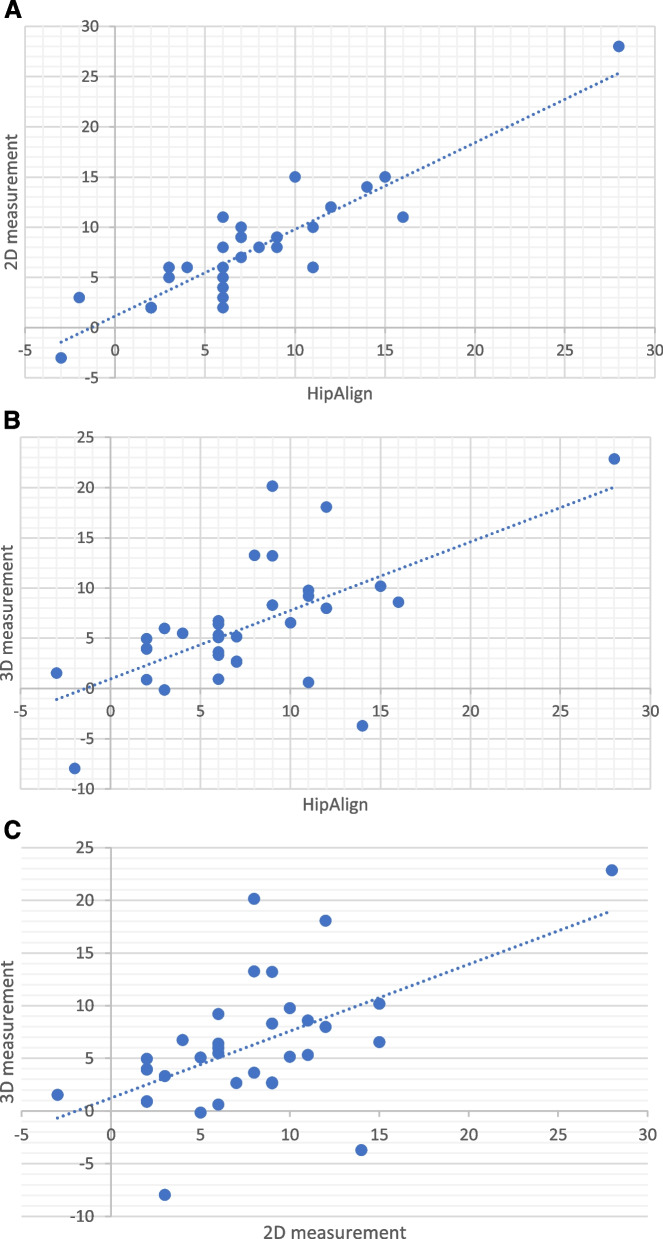
Correlations between HipAlign and 2D measurement (**A**) and 3D measurement (**B**). Correlation between 2 and 3D measurements (**C**)

The errors in leg length change for the 3D measurements were greater than those for the 2D measurements (*p* < 0.01). Positive correlations of leg length changes were found between HipAlign and 2D (*r* = 0.84, *p* < 0.01, Fig. 4A) and 3D measurements (*r* = 0.56, *p* < 0.01, Fig. 4B). Comparing the 2D and 3D measurements, there was a positive correlation with leg length change (*r* = 0.52, *p* < 0.01, Fig. 4C). Multivariate analysis demonstrated that sex was a significant contributing factor to 2D measurement errors (*p* < 0.01). Male patients demonstrated larger 2D errors. There were no significant factors for the 3D measurements (Table 1).

**Table 1:** Multivariate analyses of errors

Factor	2D	3D
Age	0.88	0.66
Sex	< 0.01	0.26
Body mass index	0.53	0.13
Approach	0.3	0.31
Crowe group	0.65	0.61

There were no early postoperative complications, including fracture, infection, and nerve injury.

## Discussion

The most significant finding of this study was that the absolute error for leg length change was greater for HipAlign evaluated with 3D measurement than 2D measurement. This confirmed our hypothesis that 2D measurement is a more accurate evaluation of leg length change when using HipAlign. An explanation for the smaller error in 2D measurement than that in 3D measurement is that HipAlign shows 2D leg length change. There was a positive correlation for leg length change between 2 and 3D measurements.

An acceptable measure of leg length discrepancy has yet to be defined. However, several studies have shown that a leg length discrepancy of up to 10 mm is well tolerated by most patients [4, 23]. Some patients, especially young patients, have complained of minor discrepancies.

Table 2 shows the results of previous reports on the accuracy of leg length measurements using navigation systems. Ogawa et al. [5] reported an absolute error of 2.4 mm with CT-based navigation. Absolute errors with portable navigation (HipAlign) were reported to be 2.3 and 3.1 mm in studies by Tanino et al. [18] and Anjiki et al. [17], respectively. The 2D errors within 5 mm were 81% and 100% in the present study and the study by Anjiki et al. [17], respectively. The accuracy of the leg length measurement in the present study was equivalent to that of previous studies, including large console navigation systems.

**Table 2:** Accuracy of leg length measurement using navigation systems

Authors	Murphy et al. [2]	Kitada et al. [3]	Ogawa et al. [5]	Renkawitz et al. [6]	Anjiki et al. [17]	Tanino et al. [18]	Present study (2D)	Present study (3D)
Navigation	CT-based			Image free	Portable			
Error (mm)	− 0.5 ± 1.8	1.3 ± 4.1		0.35	− 0.14 ± 3.6	0.8 ± 3.4	− 0.12 ± 2.6	1.5 ± 5.2
Absolute error (mm)			2.4 ± 1.7		3.1 ± 2.5	2.3 ± 2.6	1.7 ± 1.9	4.0 ± 3.5

A small reference screw is often difficult to fix to the proximal femur in patients with DAA or ALS. In our experience, the screw was often loose. The screw was not required if we could register the same point before the neck cut and after implantation. We utilized an easy technique whereby we created a small hole in the proximal femur and performed registration at this hole. Assessing intraoperative leg length change using navigation is based on the measurement of the distance between fixed points on the pelvis and the femur. However, these techniques are unreliable as they require accurate femur repositioning. Five degrees of abduction/adduction malpositioning may lead to leg length errors of up to 8 mm [24]. Using a laser enables surgeons to reposition the femur in HipAlign accurately.

Male patients in our study had larger errors when 2D measurements were used. As men generally have larger muscle volumes than women, this could explain the increase in error value; placing the registration probe on the hole was sometimes difficult without retracting the gluteus medius, gluteus minimus, and tensor fascia latae muscle in ALS and tensor fascia latae muscle and rectus femoris in DAA. Errors in leg length measurement can occur with 2D measurements when the hips have flexion contracture or are in the pelvic position. The 2D measurement was defined as the distance between the horizontal line connecting bilateral tear drops and the medial apex of the lesser trochanter. The 3D measurement was defined as the distance from the ASIS to the intercondylar fossa of the femur [19]. The importance of 3D measurement was reported in femoral offset [25]; however, the usefulness of 3D measurement in leg length change was not confirmed by comparison with 2D measurement [19]. Regarding leg length change, 2D evaluation alone is useful if accurate radiographs can be obtained [19]. Although 3D measurement of femoral offset is clinically important, 3D measurement of leg length change did not have a clinically important role.

The limitations of this study include the small sample size, the use of a new registration method, no assessment of the offset, and the use of CT. The offset is an important factor requiring intraoperative assessment, however, HipAlign cannot determine the offset intraoperatively. CT has several drawbacks, including radiation exposure and cost [26]. Recently, low-dose CT has been used for preoperative planning and postoperative assessment of THA [27].

## Conclusions

The absolute error in leg length change for the HipAlign and 3D measurement (4.03 mm) was greater than the HipAlign and 2D measurement (1.71 mm). Positive correlations between leg length changes were found between HipAlign and 2D and 3D measurements. HipAlign used in the supine position provided acceptable accuracy for leg length change measurement. 3D measurement is not recommended for the evaluation of leg length change after THA using HipAlign.

## Data Availability

The datasets during and/or analyzed during the current study are available from the corresponding author on reasonable request.

## Abbreviations

2D: Two-dimensional
3D: Three-dimensional
THA: Total hip arthroplasty
CT: Computed tomography
BMI: Body mass index
DAA: Direct anterior approach
ALS: Anterolateral supine approach
ASIS: Anterior superior iliac spine
